# Systemic inflammation in dogs with advanced-stage heart failure

**DOI:** 10.1186/s13028-018-0372-x

**Published:** 2018-03-24

**Authors:** Aleksandra Domanjko Petrič, Tajda Lukman, Barbara Verk, Alenka Nemec Svete

**Affiliations:** Small Animal Clinic, Veterinary Faculty, University of Ljubljana, Gerbičeva 60, 1000 Ljubljana, Slovenia

**Keywords:** Congestive heart failure, C-reactive protein, Dogs, Leukocytes, Neutrophils

## Abstract

**Background:**

Although human studies have shown that inflammation plays a role in the development of congestive heart failure, scarce information exists on white blood cell count (WBC) and differential cell counts in various stages of heart failure in man and dogs. A few studies demonstrated increased concentrations of C-reactive protein (CRP), a major acute-phase protein, in cardiac diseases in dogs. Our research aimed to investigate whether CRP concentration, WBC and neutrophil count (NEUT), as markers of systemic inflammation, are elevated in canine cardiovascular patients. We also aimed to find out whether there is an association between CRP concentration and WBC and NEUT, as well as associations between these inflammatory markers and selected echocardiographic parameters. Sixty-two client-owned canine cardiac patients and 12 healthy dogs were included in the study. The patients were classified into International Small Animal Cardiac Health Council classes (ISACHC I–III). The serum CRP concentration was determined using a canine CRP test kit. WBC and NEUT were determined using an automated hematology analyzer.

**Results:**

Significantly higher serum CRP concentration, WBC and NEUT were found in the decompensated stage of heart failure (ISACHC III) compared with healthy dogs and with patients in ISACHC group II and ISACHC group I. Serum CRP concentration significantly positively correlated with WBC (r = 0.65, *P* < 0.001) and NEUT (r = 0.58, *P* = 0.002) in the ISACHC III group, while no significant correlations were found in the ISACHC I and II groups. A significant negative correlation between serum CRP concentration and the left ventricular ejection fraction (r = − 0.49, *P* = 0.046) and a significant positive correlation between CRP and the E wave velocity of the mitral valve inflow (r = 0.52, *P* = 0.046) were found in the ISACHC III group.

**Conclusions:**

The CRP concentration, WBC and NEUT were significantly increased in advanced-stage heart failure patients in comparison with compensated patients and healthy dogs, which indicate the presence of systemic inflammation. However, normal CRP concentration and normal WBC and NEUT can also be present in heart failure.

**Electronic supplementary material:**

The online version of this article (10.1186/s13028-018-0372-x) contains supplementary material, which is available to authorized users.

## Background

Inflammation plays a role in the pathogenesis and progression of many forms of heart failure, and biomarkers of inflammation have become the subject of intensive investigations in human and veterinary medicine [1, 2]. In humans, C-reactive protein (CRP), measured using a high sensitivity CRP assay (hsCRP assay), as marker of inflammation has received the most attention [3–5]. Concentrations of CRP previously considered to be normal have been shown to predict future cardiovascular events independently in initially healthy individuals, as well as in human cardiovascular patients [4–8]. CRP is a major acute-phase protein in dogs that is characterized by a marked change in serum concentration consistent with systemic inflammatory activity [9–12]. It is produced primarily by the liver in response to proinflammatory cytokines, such as interleukin-1, interleukin-6 and tumor necrosis factor alpha [9, 12].

Several clinical studies have demonstrated significant alterations in some of the inflammatory markers in canine cardiovascular patients [13–18]. Increased concentrations of CRP have been demonstrated in acquired [14, 16–18] and congenital [19] cardiac diseases in dogs. In these studies, the CRP Enzyme-Linked ImmunoSorbent Assay [14, 18], automated canine-specific hsCRP assay [17] or human turbidimetric CRP assay, validated for use in dogs [16], were used for determination of CRP concentrations in serum or plasma. Moreover, in dogs with myxomatous mitral valve disease (MMVD) and dilated cardiomyopathy, plasma CRP concentration was associated with disease severity [17, 18]. However, no associations were found between serum CRP concentration, measured using a hsCRP assay, and severity of asymptomatic MMVD [17].

White blood cell count (WBC) is a marker of systemic inflammation, but data on its association with heart failure risk are limited in humans [20–22] and dogs [13, 23]. Moreover, WBC and the percentages of neutrophils, lymphocytes and eosinophils upon admission were found to be significant predictors of the development of congestive heart failure (CHF) in patients with acute myocardial infarction [24]. During years of clinical work, we observed that dogs in CHF had elevated WBC and neutrophil count (NEUT), which leads many practitioners to unjustified use of antibiotics (personal observations). Higher WBC and NEUT and markedly lower total lymphocyte count, as well as percentages of lymphocyte subpopulations in dogs in severe CHF in comparison to the control group were observed also by Farabaugh et al. [13] and Deepti and Yathiraj [23] recently found leukocytosis in CHF dogs.

We hypothesize that canine heart failure patients have elevated markers of systemic inflammation (CRP, WBC and NEUT). Components of the inflammatory process contribute to the worsening and progression of heart failure and imbalance of regulatory mechanisms that maintain cardiac homeostasis [25, 26]. Our research aimed 1) to investigate whether CRP concentration, WBC and NEUT, as markers of systemic inflammation, are elevated in canine cardiovascular patients; 2) to find out whether there is an association between CRP concentration and WBC and NEUT, as well as associations between these inflammatory markers and selected echocardiographic parameters.

## Methods

### Animals

Eighty-one client-owned dogs of various breeds, sex, age and weight were recruited. Sixty-nine dogs were cardiovascular patients prospectively recruited, and 12 healthy dogs served as controls. The 12 control dogs were from the active rescue dog team and were judged to be healthy based upon normal history, normal clinical examination and the results of hematological and biochemical analyses. All dogs included in the study were regularly dewormed and vaccinated and none of the dogs showed eosinophilia indicative of a parasitic or allergic condition.

Cardiovascular disease was confirmed on the basis of history, clinical examination, radiographic examination of the thorax, standard electrocardiogram and echocardiography using two-dimensional, M-mode, and color and spectral Doppler modes (Vingmed System Five, General Electric Healthcare, Milwaukee, Wisconsin, USA). Nine-lead electrocardiogram was performed in the right lateral recumbence or in standing position, depending of the dog’s status. Echocardiography was done according to the recommendations [27]. Left ventricular internal dimensions in diastole and systole were indexed to body weight in kg raised by the exponent according to Cornell et al. [28]. Left ventricular ejection fraction was calculated by the Teichholz method derived from M-mode measurements. Echocardiographic parameters, including left atrium dimension and diameter of the aorta ratio (LAD/Ao), percentage fractional shortening (pctFS), left ventricular dimension at end diastole index (LVDdI), left ventricular ejection fraction (LVEF), left ventricular dimension at end systole index (LVDsI) and E wave velocity of the mitral valve inflow (MVE) were used for correlation analysis to find out whether there is an association between these parameters and inflammatory markers. Dogs with concomitant non-cardiac diseases, including other diseases such as neoplasia, infections or metabolic disorders, were excluded based on the results of hematologic and biochemical analyses, ancillary laboratory tests (urinalysis, *Anaplasma phagocytophilum* (serologic and polymerase chain reaction assay), *Borrelia burgdorferi* serological testing) and abdominal ultrasound. Patients were classified into the International Small Animal Cardiac Health Council (ISACHC) classes [29]. Asymptomatic dogs with documented cardiac disease were included in ISACHC I; however, this class was not further divided. In the ISACHC II group we included dogs with documented cardiac disease and compensated, i.e. well controlled heart failure with cardiac therapy and no overt clinical signs of decompensation. In compensated heart failure, symptoms are stable and many overt features of fluid retention and pulmonary edema are absent. In the ISACHC III group, we included dogs that were in uncontrolled/decompensating heart failure at the time of inclusion. Decompensated heart failure refers to deterioration, which may present either as an acute episode of pulmonary edema or as lethargy and reduction in exercise tolerance and increasing tachypnea/dyspnea on exertion. These later dogs were either first time patients without CHF therapy or worsening of already diagnosed CHF. Criteria for the diagnosis of CHF were based on clinical signs and subjectively assessed thoracic radiography. An interstitial or alveolar pulmonary pattern compatible with pulmonary edema with left atrial enlargement and clinical signs of CHF were used to confirm CHF.

Written consent of the owners was obtained. All procedures were approved by the Ethical Committee of the Ministry of Agriculture, Forestry and Food, Veterinary Administration of the Republic of Slovenia (Animal Protection Act UL RS 43/2007).

### Blood sampling and analysis

Blood was taken from the jugular or cephalic vein from fasted dogs. Blood samples for determining complete blood count (CBC) and white cell differential count (WCDC) were collected into tubes containing the anticoagulant ethylenediaminetetraacetic acid (BD Microtainer; Becton–Dickinson, Franklin Lakes, New Jersey, USA). Blood samples for determining the biochemical profile (urea, creatinine, electrolytes (sodium, potassium and chloride), alkaline phosphatase, and alanine aminotransferase; data not shown) and CRP concentrations were collected in serum separator tubes (Vacuette; Greiner Bio-One, Kremsmünster, Austria). Blood samples were centrifuged for 10 min at 1300×*g*. CBC and WCDC were determined within 1 h after collection of the blood samples using an automated laser-based hematology analyzer (ADVIA 120, Siemens, Munich, Germany) and multispecies software, developed by the manufacturer. All settings in the software were used as provided, without adjustment or modification.

The biochemical profiles, with the exception of electrolytes, were determined with an automated biochemical analyzer (RX Daytona, Randox, Crumlin, United Kingdom) on the day of blood sample collection. The concentrations of electrolytes were determined with an electrolyte analyzer (ILyte, Instrumentation Laboratory, Lexington, MA, USA) on the day of sample collection. Serum samples for CRP measurements were frozen at − 80 °C and analyzed within 2 months. Serum CRP concentrations were determined with the canine CRP test kit (LifeAssays^®^ Canine CRP test kit, LifeAssays®, Lund, Sweden), which is a one-step 11-min magnetic permeability based canine-specific two-site immunoassay [30, 31]. The linear range of the test kit is 10–210 mg/L. All samples were analyzed in duplicate and the mean CRP concentration was used for statistical analyses. The canine CRP control, included in the canine CRP test kit, was used to confirm the efficacy of the reagents and correct performance of the test. The control samples were analyzed according to the same procedure as for a canine sample. The detection limit (DL) of the assay was 10 mg/L. Therefore, the samples with CRP concentration of less than the DL of the assay were assigned a concentration of 10 mg/L and for statistical analysis used as such or treated with special methods.

### Statistical analysis

The data were analyzed with commercial software (SPSS version 22.0, SPSS Inc, Chicago, Illinois, USA and R statistical program [32]. A value of *P* < 0.05 was considered significant. Descriptive statistics was used to describe the basic features of the data. The Shapiro–Wilk test was performed to test whether the data were normally distributed. A one-way ANOVA with Tukey Honest Significant Difference post hoc test was used to test for statistically significant differences in age between healthy dogs and individual groups of cardiac patients (ISACHC I–III). Kruskal–Wallis analysis followed by multiple comparisons was performed to compare the measured parameters (WBC, NEUT), as well as weight, between healthy dogs and individual groups of cardiac patients (ISACHC I–III). For the comparison of CRP (ISACHC I–III) a two-part model combining χ^2^ and Kruskal–Wallis test [33] was used to account for the values below DL (see supplement). To control for the confounding variables (age, sex and disease etiology) two multiple linear regression models for (log_10_)WBC and (log_10_)NEUT each were performed (disease etiology could be controlled for only in the subset of diseased patients). A two-part model combining logistic and linear regression on diseased patients was used to account for the values below DL in CRP.

Spearman’s rank correlation coefficient analysis was performed to determine the correlation between the CRP concentration and WBC and NEUT, as well as selected echocardiographic parameters (LAD/Ao, pctFS, LVDdI, LVEF, LVDsI, and MVE). The same method was used to determine correlations between CRP concentrations and the intensity of cardiac murmur grades 1–6.

## Results

Seven cardiovascular patients were excluded from the group of 69 patients, due to various reasons: antibiotic therapy and dental disease (2 dogs), allergy, hypothyroidism, antibiotic therapy with suspected endocrine disorder, cachexia and lost blood sample. Sixty-two dogs of 25 different breeds with confirmed cardiovascular diseases were divided in three groups according to the ISACHC classification, based on a complete cardiovascular examination. Medication regimens included diuretics (furosemide and spironolactone), beta-blockers (atenolol), calcium-channel blockers (diltiazem), ACE (angiotensin-converting enzyme) inhibitors, an inodilator (pimobendan), and digoxin (Table 1). Cardiovascular patients were of the following breeds: Mixed breed dogs (n = 16), Doberman Pinschers (n = 5), Cavalier King Charles (n = 5), 4 Chihuahuas (n = 4), Great Danes (n = 3), German Boxers (n = 3), Italian Greyhounds (n = 3), Airedale Terriers (n = 2), Rottweilers (n = 2), Saint Bernard dogs (n = 2), German Shepherds (n = 2), Labrador Retrievers (n = 2) and one dog of each breed: Pekingese, Japanese Chin, Shih-Tzu, Newfoundland Dog, Boston Terrier, Miniature Poodle, Tibetan Terrier, Rhodesian Ridgeback, Beagle, Dogue de Bordeaux, Dachshund, American Staffordshire Terrier and Schipperke. Twelve healthy dogs were enrolled as controls in the study. The following breeds were included in the control group: Labrador Retrievers (n = 6), Golden Retrievers (n = 3), Mixed breed dogs (n = 2), and Border Collie (n = 1). Dog characteristics other than breed are summarized in Table 2.

**Table 1:** Treatment used in groups of canine cardiac patients (ISACHC I–III)

Therapeutics	ISACHC I (N = 10) (*n*)	ISACHC II (N = 26) (*n*)	ISACHC III (N = 26) (*n*)
Salbutemol	1		
Furosemide		26	21
ACE inhibitor		26	13
Pimobendan	1	15	14
Atenolol (beta-blocker)		2	4
Digoxin		5	9
Diltiazem (calcium-channel blocker)		4	4
Spironolactone		4	1
Potassium chloride		5	
Total number of dogs with therapy	2	26	21

*ISACHC* International Small Animal Cardiac Health Council; *N* number of dogs included in ISACHC group; *n* number of dogs per ISACHC group receiving specific cardiac drug; *ACE* angiotensin-converting enzyme

**Table 2:** Baseline characteristics in healthy dogs and groups of canine cardiac patients (ISACHC I–III)

Group	Healthy	ISACHC I	ISACHC II	ISACHC III
Number	12	10	26	26
Sex (female/male)	9/3	3/7	8/18	7/19
Age (years) mean ± SD	6.7 ± 3.4	7.3 ± 3.7	10.8 ± 3.9^*^	10.3 ± 4.1^*^
Weight (kg)				
Median	28.7	14.0	9.9	26.4
Interquartile range	22.5–33.0	6.2–45.7	6.9–36.0	10.5–32.8

*ISACHC* International Small Animal Cardiac Health Council; *SD* standard deviation

^*^ *P* < 0.05 in comparison with healthy dogs

Cardiovascular patients had the following diseases: myxomatous mitral valve disease (MMVD; ISACHC I, n = 6; ISACHC II, n = 19; ISACHC III, n = 14), dilated cardiomyopathy (ISACHC I, n = 2; ISACHC II, n = 5; ISACHC III, n = 9), subaortic stenosis (ISACHC I, n = 2) and patent ductus arteriosus (ISACHC II, n = 2; ISACHC III, n = 3).

**Fig. 1:**
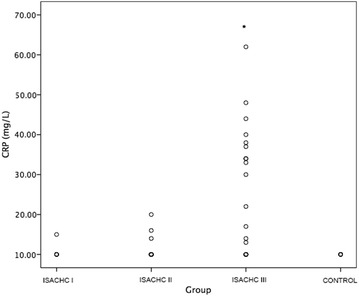
Serum CRP concentrations in healthy dogs and groups of canine cardiac patients (ISACHC I–III). *significantly higher in comparison with ISACHC II (*P* < 0.001) and ISACHC I (*P *= 0.011) and healthy dogs (*P* = 0.001)

**Fig. 2:**
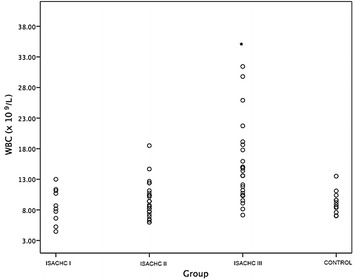
White blood cell count in healthy dogs and groups of canine cardiac patients (ISACHC I-III). *significantly higher in comparison with ISACHC II (*P *< 0.001) and ISACHC I (*P* = 0.004) and healthy dogs (*P* = 0.005)

**Fig. 3:**
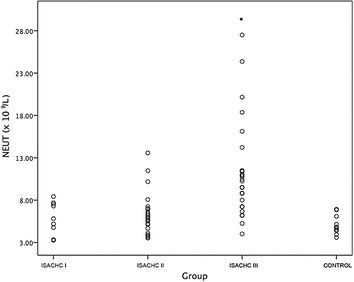
Neutrophil count in healthy dogs and groups of canine cardiac patients (ISACHC I-III). *significantly higher in comparison with ISACHC II (*P* < 0.001) and ISACHC I (*P* = 0.003) and healthy dogs (*P* < 0.001)

A significantly higher serum CRP concentration (Fig. 1) was found in the decompensated stage of heart failure (ISACHC III; median 15.5 mg/L, interquartile range 10.0–34.8 mg/L) compared with healthy dogs (*P* = 0.001; median 10.0 mg/L, interquartile range 10.0–10.0 mg/L) and with patients in ISACHC group II (*P* < 0.001; median 10.0 mg/L, interquartile range 10.0–10.0 mg/L) and ISACHC group I (*P* = 0.011; median 10.0 mg/L, interquartile range 10.0–10.0 mg/L). We found a significantly higher WBC (Fig. 2) in ISACHC III dogs (median 14.0 × 10^9^/L, interquartile range 10.4–18.0 × 10^9^/L) compared with healthy dogs (*P* = 0.005; median 9.0 × 10^9^/L, interquartile range 7.8–10.2 × 10^9^/L), ISACHC I dogs (*P* = 0.004; median 8.4 × 10^9^/L, interquartile range 6.3–11.2 × 10^9^/L) and ISACHC II dogs (*P* < 0.001; median 9.1 × 10^9^/L, interquartile range 7.6–10.8 × 10^9^/L). In addition, a significantly higher NEUT (Fig. 3) was observed in ISACHC III dogs (median 10.4 × 10^9^/L, interquartile range 7.2–12.2 × 10^9^/L) compared with healthy dogs (*P* < 0.001; median 4.8 × 10^9^/L, interquartile range 4.4–5.9 × 10^9^/L), ISACHC I dogs (*P* = 0.003; median 5.5 × 10^9^/L, interquartile range 3.3–7.6 × 10^9^/L) and ISACHC II dogs (*P* < 0.001; median 6.0 × 10^9^/L, interquartile range 4.5–7.0 × 10^9^/L).

In the multivariate analyses, where we controlled for age, sex (and disease), ISACHC group III still had significantly larger expected values of all the three inflammatory parameters (log_10_WBC, log_10_NEUT or CRP) compared to ISACHC group II (*P* < 0.001). Expected values of inflammatory parameters for ISACHC group I in the multivariate model were never significantly different from the expected values for inflammatory parameters in group II (see Additional file 1).

Serum CRP concentration significantly positively correlated with WBC (r = 0.65, *P* < 0.001) and NEUT (r = 0.58, *P* = 0.002) in the ISACHC III group, while no significant correlations were found in ISACHC I and II groups. There were no statistically significant correlations between the serum CRP concentration and heart murmur intensity (grades 1 to 6) in any of the ISACHC groups. A significant negative correlation between serum CRP concentration and LVEF (r = − 0.49, *P* = 0.046) and a significant positive correlation with MVE (r = 0.52, *P* = 0.046) were found in the ISACHC III group. There were no significant correlations between the CRP concentration and other selected echocardiographic parameters in any of the ISACHC groups.

## Discussion

The present study is the first to report on serum CRP concentration in association with WBC and neutrophil count in patients with severe heart failure. Our results demonstrate that systemic inflammation may be present in patients with severe heart failure, irrespective of the etiology. The role of inflammation in the development and progression of heart failure has been described [15, 34–37]. Inflammation can contribute not only to myocardial dysfunction but also to detrimental consequences such as endothelial dysfunction and cardiac cachexia. Inflammatory mediators may be released from the failing myocardium itself, and also from circulating leukocytes, blood platelets, endothelial cells, and from the liver and lungs [36]. Acute phase proteins as markers of inflammation, including CRP, have been found to be increased in acute and chronic inflammatory diseases and heart failure [10, 17, 38–42].

Our study showed a significantly higher serum CRP concentration in ISACHC III heart failure patients in comparison with ISACHC II, ISACHC I and healthy dogs, although the median value of CRP concentration in ISACHC III group did not exceed the upper value of reference range [31] and more than a half of the patients in this group had CRP concentrations within the reference range. Regardless the method for measurement of circulating CRP concentration used, recent studies have reported significantly higher serum CRP concentration in dogs with CHF due to MMVD or dilated cardiomyopathy compared to clinically healthy dogs [17, 18] or in comparison to asymptomatic dogs [16, 17], which is in accordance with our results. Similarly, in the study of Reimann et al. [17], CRP was not increased in dogs with different stages of MMVD without CHF in comparison with control dogs; however, the presence of CHF significantly increased serum CRP concentration. Contrary to our results, Rush et al. [14] found no significant difference in CRP concentration between MMVD dogs with CHF and without CHF; however, CRP concentration was significantly higher in dogs with MMVD (with and without CHF) in comparison with healthy dogs. The differences between the results of our study and that of Rush et al. [14] might be attributed to differences in dogs included in studies and different CRP assays used. Ljungvall et al. [43] found no significant differences in CRP concentration between healthy dogs and dogs at different stages of MMVD that were not in CHF. It has been suggested that renin–angiotensin–aldosterone system with sympathetic system contribute to constant inflammation in CHF. Studies have shown that angiotensin II activates leukocytes in circulation and plays a role in their adhesion to the endothelium. Additionally, lymphocytes and monocytes express beta-adrenergic receptors and beta-adrenergic stimulation may modulate cytokine production in these cells [37]. Our results may indicate that the progression of CHF is the major cause of increased levels of systemic markers of inflammation. Studies with human CHF patients have revealed that WBC and CRP may be implicated in the development of heart failure by the immune system acting as a modulator of myocyte injury and inflammatory reactions contributing to the structural and functional deterioration observed in failing human hearts [44]. Additionally, CRP may be an independent marker of improvement and readmission in heart failure [45]. Higher levels of plasma CRP concentration, measured using a hsCRP assay, have been associated with more severe heart failure and independently associated with morbidity and mortality [6, 39]. Our study revealed a significant positive correlation between CRP and MVE in ISACHC III, which indicates that higher serum CRP concentrations are associated with higher left atrial pressure and thus more severe CHF. Moreover, CRP concentration significantly negatively correlated with LVEF, which additionally supports our results that higher CRP concentrations are associated with more severe CHF and myocardial dysfunction. On the other hand, we did not find a significant correlation between murmur grade and CRP concentration in any of the ISACHC groups. Similarly, Rush et al. [14] found no significant correlation between murmur grade and CRP concentrations in dogs with chronic valvular disease.

In our study, CRP concentration significantly positively correlated with WBC and NEUT in the ISACHC III group. In this group of patients, WBC and NEUT were significantly higher compared with that in ISACHC II and ISACHC I and healthy dogs. In the ISACHC III group, the median values of WBC and NEUT exceeded the upper value of reference ranges in dogs [46], indicating leukocytosis and neutrophilia in CHF dogs, which suggests that systemic inflammation may be present in advanced stage of heart failure. In addition, authors believe that WBC needs to be judged on an individual basis and not blindly compared to the reference values. Farabaugh et al. [13] found significantly higher WBC and NEUT in CHF dogs (ISACHC III) compared with ISACHC II and the control group, which is in accordance with our results. Deepti and Yahtiraj [23] also found higher mean WBC in CHF dogs compared to the reference values from the literature; however, they did not have their own control group. Few human studies have demonstrated a correlation between WBC and CHF. In human patients with acute myocardial infarction, increased levels of WBC and NEUT and decreased levels of lymphocytes and eosinophils were found to be associated with early development of CHF [24]. Higher incidence of in-hospital deaths of human patients due to decompensated heart failure were found to be associated with higher CRP concentration, higher leukocyte and neutrophil counts and lower lymphocyte count, which supports our findings [47]. A high number of leukocytes may affect the electrical stability of the heart. It has been shown that high WBC is a significant predictor of ventricular fibrillation in patients with acute myocardial infarction [20]. Additionally, high WBC have been associated with the development of new CHF or shock [20]. Mechanisms by which leukocytes may affect the progression of CHF include proteolytic damage, leukocyte aggregation, microvascular obstruction, electrical instability and impaired revascularization [20, 24, 48]. Neutrophils may not directly contribute to contractile dysfunction but may be an indicator of other inflammatory mediators that may be directly involved in the pathogenesis of cardiac dysfunction. Moreover, neutrophils as phagocytes undergo a cellular respiratory burst and release free oxygen radicals, which are toxic to cells. Interaction between neutrophils and inflammatory cells within the myocardium can occur, which results in the release of lysosomal enzymes and arachidonic acid metabolites, thus causing myocardial dysfunction [49–51].

Our study has some limitations. These include quite high DL (10 mg/L) of the canine CRP test used, which disables more sensitive determination of CRP concentration. However, hsCRP assays with much lower detection limits were unable to discriminate between degrees of MMVD without CHF but were able to detect the significant difference in CRP concentration between patients with and without CHF [17], as well as between CHF patients and healthy dogs [17, 18]. The same results were obtained by the CRP test used in our study. In a clinical setting, it is important to recognize that systemic inflammation may be present in acute decompensated heart failure of already treated or new heart failure patients in order not to interpret the results as infection. We would like to point out that practitioners should consider CHF as a differential diagnosis in case of elevated CRP, WBC and NEUT. In this case irrational use of antibiotics can be avoided. The CRP assay used in our study is widely available and may be used by a practitioner because it is easy to use, rapid (it takes only 11 min to get the result) and does not require trained laboratory personnel. In cases of CRP concentrations below the detection limit, CHF needs to be considered in patients with typical clinical signs that may include tachypnea/dyspnea, cough, tachycardia, a heart murmur, a weak femoral pulse and/or arrhythmias. For confirmation of CHF, additional, more specific methods should be used (N-terminal pro-B-type natriuretic peptide, thoracic radiography and echocardiography). Another limitation could be relatively low number of dogs included in the study and the inclusion of more than one heart disease into CHF classes; however, the multiple regression analysis showed that etiology had no effect on the results presented. Another limitation might be that the control dogs had no echocardiographic examination done; however, these were all active rescue dogs without any clinical signs and blood-work abnormalities. In addition, the control group was not breed matched with patient groups; majority of control dogs consisted mostly of large breeds. Control dogs were also significantly younger compared to ISACHC II and III groups; however, no significant age-related differences in CRP concentrations were observed in healthy beagle dogs [52]. Moreover, no associations between CRP and age, body weight or breed were found in canine cardiac patients [18]. In humans, CRP concentration increased with age; however, it remained within the normal range [53]. We cannot totally exclude possible effect of age and breed on hematological variables [54]. Another limitation might be the fact that elderly dogs may have a higher prevalence of comorbidities (periodontal disease, osteoarthritis) that may result in increased values of inflammatory markers [55, 56]; however, dogs with advanced periodontal disease and symptomatic osteoarthritis were not included in this study. The majority of our patients had therapy, which may influence the inflammatory markers [57–59]. In humans, neutrophilia and lymphopenia were more pronounced in CHF patients that were not taking beta-blockers versus those taking this treatment [57]. Another human study showed that administration of specific beta-blockers could be associated with attenuation of inflammation [58]. Short-term inotropic support reduced indices of inflammation in patients with decompensated CHF [59].

This study demonstrated that irrespective of etiology, CHF may be associated with inflammatory process, as evident from significantly increased levels of inflammatory markers (CRP concentration, WBC and NEUT) in decompensating or severe heart failure in comparison with compensated patients and healthy dogs; however, normal CRP concentration and normal WBC/NEUT can also be present in heart failure. It is worth noting that all three measured parameters show an increasing trend with the stage of cardiac disease. Our results give an additional evidence of the inflammatory nature of severe heart failure in dogs as it was already documented with the use of CRP and cytokines in humans [34–36, 42] and dogs [15, 17, 18]. Practitioners should be aware of the presence of inflammation when interpreting complete blood count, white cell differential count or CRP concentration in canine cardiovascular patients.

## Conclusions

The study reported here demonstrated significantly increased levels of CRP and WBC and NEUT in dogs with severe, i.e. decompensated CHF in comparison with compensated patients and healthy dogs. However, normal CRP concentration and normal WBC and NEUT can also be present in heart failure. Furthermore, in the group of patients with severe CHF, a significant association between CRP concentration and WBC and NEUT was found. Our results support the hypothesis that systemic inflammation may be present in patients with severe heart failure. This information may help also the practitioners to be aware that systemic inflammation may be present in heart failure in order not to interpret the results of WBC, NEUT and CRP as infection; however, it should be kept in mind that these markers are unspecific and should be interpreted with the history and clinical signs as well as other more specific diagnostic modalities.

## Additional file

**Additional file 1.** Procedures for the multivariate models.

## Abbreviations

ACE: angiotensin-converting enzyme
ANOVA: analysis of variance
CBC: complete blood count
CHF: congestive heart failure
CRP: C-reactive protein
DL: detection limit
hsCRP: high sensitivity C-reactive protein
ISACHC: International Small Animal Cardiac Health Council
LAD/Ao: left atrium dimension and diameter of the aorta ratio
LVDdI: left ventricular dimension at end diastole index
LVDsI: left ventricular dimension at end systole index
LVEF: left ventricular ejection fraction
MMVD: myxomatous mitral valve disease
MVE: E wave velocity of the mitral valve inflow
NEUT: neutrophil count
pctFS: percentage fractional shortening
SD: standard deviation
