# Supplementary material for: Systemic inflammation in dogs with advanced-stage heart failure
Source: Acta Vet Scand. 2018 Mar 24;60:20. doi: 10.1186/s13028-018-0372-x (PMC5866510; doi:10.1186/s13028-018-0372-x)
Supplement: Supplementary file 1 — Additional file 1. Procedures for the multivariate models. [file 13028_2018_372_MOESM1_ESM.doc]

# Additional file 1. Procedures for the multivariate models

We fitted two multiple linear regression models for the two inflammatory parameters (WBC, NEUT) in order to take the measured confounding variables into account when comparing different groups of diseased/control patients. Since WBC and NEUT are asymmetric log_10_ values were used for the outcome of the regression models.

The first regression model (Model 1) was constructed for the whole dataset. The predictors for the inflammatory parameters were group (ISACHC I, II, III or controls), age and sex (the last two as the confounding variables).

Disease (etiology of heart failure) could be included in the regression model (Model 2) for only diseased patients since disease and controls are too correlated to be included in the same model.

Inflammatory parameter CRP is specific due to its detection limit (10 mg/L). A two-part model combining logistic and linear regression was used to account for the values below detection limit. Since controls differ from the diseased (all the values of CRP are below detection limit) statistical model was fitted just for the subset of diseased (Model 2).

## (log_10_)WBC

### Model 1 – whole dataset

|  | Estimate | Std. Error | t-value | *P*-value |
| --- | --- | --- | --- | --- |
| (Intercept) | 0.962 | 0.054 | 17.963 | 0.000 |
| Group I vs. control | -0.054 | 0.061 | -0.875 | 0.385 |
| Group II vs. I | 0.030 | 0.054 | 0.559 | 0.578 |
| Group III vs. II | 0.184 | 0.038 | 4.815 | 0.000 |
| Age | 0.005 | 0.005 | 1.056 | 0.295 |
| Sex (female) | -0.022 | 0.040 | -0.554 | 0.581 |

Despite the inclusion of age and sex into the model (therefore controlling for age and sex), group III still has statistically significantly larger expected values of log_10_WBC compared to group II (*P* < 0.001). Group II does not exhibit statistically significantly different expected values in log_10_WBC as group I (*P* = 0.578) and similar is observed for the comparison between group I and the controls (*P* = 0.385).

Other included variables do not show any statistically significant linear association with log_10_WBC.

### Model 2 — subset of diseased

|  | Estimate | Std. Error | t-value | Pr (>| t|) |
| --- | --- | --- | --- | --- |
| (Intercept) | 0.978 | 0.072 | 13.680 | 0.000 |
| Group II vs. I | 0.076 | 0.060 | 1.266 | 0.211 |
| Group III vs. II | 0.190 | 0.039 | 4.819 | 0.000 |
| Age | 0.004 | 0.007 | 0.560 | 0.578 |
| MMVD vs. DCM | -0.007 | 0.048 | -0.147 | 0.884 |
| PDA vs. DCM | -0.170 | 0.090 | -1.893 | 0.064 |
| SAS vs. DCM | 0.144 | 0.125 | 1.152 | 0.254 |
| Sex (female) | -0.002 | 0.049 | -0.033 | 0.974 |

Controlling for age, sex and disease, group III shows statistically significantly larger expected values of log_10_WBC compared to group II (*P* < 0.001). Group II does not exhibit statistically significantly different expected values in log_10_WBC as group I and the same for the comparison between group I and the controls; MMVD: myxomatous mitral valve disease; DCM: dilated cardiomyopathy; PDA: patent ductus arteriosus; SAS: subaortic stenosis.

.

## (log_10_)NEUT

### Model 1 – whole dataset

|  | Estimate | Std. Error | t value | Pr (>| t|) |
| --- | --- | --- | --- | --- |
| (Intercept) | 0.753 | 0.063 | 11.920 | 0.000 |
| Group I vs. C | 0.015 | 0.072 | 0.214 | 0.831 |
| Group II vs. I | 0.023 | 0.063 | 0.363 | 0.717 |
| Group III vs. II | 0.239 | 0.045 | 5.302 | 0.000 |
| Age | 0.007 | 0.006 | 1.236 | 0.221 |
| Sex (female) | -0.030 | 0.047 | -0.641 | 0.524 |

Controlling for age and sex, group III shows statistically significantly larger expected values of log_10_NEUT compared to group II (*P* < 0.001). Group II does not exhibit statistically significantly different expected values in log_10_NEUT as group I and the same for the comparison between group I and the controls.

Other variables do not show any statistically significant linear association with log_10_NEUT.

### Model 2 — subset of diseased

|  | Estimate | Std. Error | t value | Pr (>| t|) |
| --- | --- | --- | --- | --- |
| (Intercept) | 0.794 | 0.085 | 9.321 | 0.000 |
| Group II vs. I | 0.079 | 0.072 | 1.102 | 0.275 |
| Group III vs. II | 0.244 | 0.047 | 5.197 | 0.000 |
| Age | 0.006 | 0.009 | 0.712 | 0.480 |
| MMVD vs. DCM | -0.022 | 0.057 | -0.376 | 0.709 |
| PDA vs. DCM | -0.203 | 0.107 | -1.895 | 0.063 |
| SAS vs. DCM | 0.175 | 0.149 | 1.172 | 0.246 |
| Sex (female) | 0.002 | 0.058 | 0.029 | 0.977 |

Controlling for age, sex and disease group III shows statistically significantly larger expected values of log_10_NEUT compared to group II (*P* < 0.001). Group II does not exhibit statistically significantly different expected values in log_10_NEUT as group I and the same for the comparisons between group I and the controls; MMVD: myxomatous mitral valve disease; DCM: dilated cardiomyopathy; PDA: patent ductus arteriosus; SAS: subaortic stenosis.

## CRP

## Model 2 — subset of diseased

|  | OR | P-value logistic regression | B | P-value linear regression overall | *P*-value |
| --- | --- | --- | --- | --- | --- |
| (Intercept) | 0.181 | 0.253 | 16.386 | 0.231 | 0.234 |
| Group II vs. I | 0.801 | 0.871 | 2.474 | 0.876 | 0.974 |
| Group III vs. II | 12.173 | 0.002 | 16.571 | 0.077 | 0.002 |
| Age | 1.215 | 0.212 | 0.827 | 0.554 | 0.381 |
| Sex (female) | 0.161 | 0.145 | 13.477 | 0.358 | 0.219 |
| MMVD vs. DCM | 0.136 | 0.042 | -10.342 | 0.230 | 0.056 |
| PDA vs. DCM | 1.707 | 0.780 | -10.008 | 0.672 | 0.875 |
| SAS vs. DCM | 0.000 | 0.994 |  |  |  |

Controlling for age, sex and disease in the subset of diseased patients, group III shows statistically significantly larger odds for CRP above detection limit (OR = 12.2, *P* = 0.002) and a nearly significant increase in CRP for the detected values (coefficient B = 16.6, *P* =0.077). These two results are combined in a significant effect with *P* = 0.002. There exist statistically significant differences in the distribution of CRP values between the two groups (III vs. II). Other comparisons do not show any statistically significant differences; MMVD: myxomatous mitral valve disease; DCM: dilated cardiomyopathy; PDA: patent ductus arteriosus; SAS: subaortic stenosis.
